# Estimating the prevalence of mental disorders in patients with newly diagnosed cancer in relation to socioeconomic status: a multicenter prospective observational study

**DOI:** 10.1016/j.esmoop.2024.103655

**Published:** 2024-07-31

**Authors:** U. Goerling, J. Ernst, P. Esser, C. Haering, M. Hermann, B. Hornemann, P. Hövel, U. Keilholz, D. Kissane, O. von dem Knesebeck, F. Lordick, F. Springer, H. Zingler, T. Zimmermann, C. Engel, A. Mehnert-Theuerkauf

**Affiliations:** 1 Charité—Universitätsmedizin Berlin, Corporate Member of Freie Universität Berlin, Humboldt-Universität zu Berlin, and Berlin Institute of Health, Charité Comprehensive Cancer Center, Berlin; 2 Department of Medical Psychology and Medical Sociology, Comprehensive Cancer Center Central Germany (CCCG), University Medical Center Leipzig, Leipzig; 3 Comprehensive Cancer Center, University Clinic Centre Dresden, Dresden, Germany; 4 School of Medicine, University of Notre Dame Australia, Sydney; 5 Departments of Palliative Care, Cabrini Health, Melbourne; 6 Department of Palliative Care, St Vincent’s Hospital Sydney, Sydney; 7 School of Clinical Sciences, Monash Health and Monash University, Melbourne, Australia; 8 Institute of Medical Sociology, University Medical Center Hamburg-Eppendorf, Hamburg; 9 Department of Medicine II (Oncology, Gastroenterology, Hepatology, and Pulmonology), Comprehensive Cancer Center Central Germany (CCCG), University of Leipzig Medical Center, Leipzig; 10 Department of Psychosomatic Medicine and Psychotherapy, Hannover Medical School, Hannover; 11 Institute for Medical Informatics, Statistics and Epidemiology, Leipzig University, Leipzig, Germany

**Keywords:** quality of life, supportive care, symptom management

## Abstract

**Background:**

The purpose of this study was to provide the 4-week prevalence estimates of mental disorders in newly diagnosed cancer patients in relation to socioeconomic status (SES).

**Patients and methods:**

We enrolled newly diagnosed patients with a confirmed solid tumor within 2 months of diagnosis. We calculated patients’ SES on the basis of their educational level, professional qualification, income and occupational status. We used the Structured Clinical Interview for Diagnostic and Statistical Manual of Mental Disorders, Fifth Edition—Clinical Version (SCID-5-CV) to assess the 4-week prevalence of mental disorders in addition to a comorbidity questionnaire to assess the level of physical impairment.

**Results:**

We identified a total of 1702 patients with mixed cancers after reviewing their medical records and contacting them in person or by post due to coronavirus pandemic patient safety restrictions. 1030 patients (53.2% men, mean age 60.2 years) had completed SCID-5-CV. When weighted according to the SES distribution to account for over- and under-sampling of SES groups, 20.9% [95% confidence interval (CI) 18.1% to 23.6%] of patients were diagnosed with any mental disorder. The most prevalent were depressive disorders (9.9%, 95% CI 7.9% to 11.9%), trauma and stress-related disorders (6.3%, 95% CI 4.7% to 7.9%) and anxiety disorders (4.2%, 95% CI 2.9% to 5.6%). We found no difference in any mental disorder between patients with high, medium or low SES. Multivariate logistic regression analyses revealed higher proportion of patients with any mental disorder in patients younger than 60 years [odds ratio (OR) 0.42; *P* < 0.001], in patients without a partner (OR 1.84; *P* < 0.001), in women with tumor in female genital organs (OR 2.45; *P* < 0.002) and in those with a higher level of impairment (OR 1.05, 95% CI 1.03-1.07; *P* < 0.001).

**Conclusions:**

SES had no significant influence on mental comorbidity in early cancer survivorship.

## Highlights

• A 4-week SES-weighted prevalence of 20.9% for any mental disorder was found in newly diagnosed cancer patients.
• There was no significant difference in any mental disorder between patients with high, medium or low SES.
• Younger age, lack of a partner, tumor in female genital organs and higher level of impairment may predict mental disorder.
• Two vulnerable groups with regard to higher mental comorbidity: younger patients with low SES and men with low SES.

## Introduction

The interaction between physical and mental health conditions, behavioral and social factors that influence morbidity and quality of life both at the onset and during the course of disease is a multifaceted and increasingly researched area in medicine and psychology. The increasing incidence of cancer will lead to a higher prevalence of cancer survivors as there are remarkable improvements in cancer research, diagnostics and treatments, turning many cancers into chronic diseases.^1^ At the same time, the psychological burden on patients is known to be high, due to the short-, middle-, long-term and late effects of the disease and treatment, but also due to pre-existing psychosocial vulnerabilities.^2^, ^3^, ^4^, ^5^, ^6^, ^7^, ^8^, ^9^ The meta-analysis by Mitchell et al.^10^ of over 70 studies shows a pooled prevalence rate of 19.4% for adjustment disorders, 16.3% for depression and 10.3% for anxiety disorders. Our previous epidemiological study in Germany with >4000 cancer patients found a 4-week prevalence of 31.8% for overall mental disorders, with anxiety disorders (11.5%) and adjustment disorders (11.1%) being the most common according to the Diagnostic and Statistical Manual of Mental Disorders, Fourth Edition (DSM-4).^11^

Mental comorbidity in cancer populations has been associated with female gender, poor physical function, age, pain and lower social support.^2^^,^^12^, ^13^, ^14^, ^15^ However, data on the association between socioeconomic status (SES) and mental comorbidity in cancer populations are insufficient, although it has been extensively demonstrated for other diseases.^16^, ^17^, ^18^, ^19^, ^20^, ^21^, ^22^ This is important, as poor mental health is associated with numerous adverse outcomes, including poor quality of life, higher morbidity, worse survival and higher mortality^23^, ^24^, ^25^, ^26^ and higher costs for the health system.^27^ Mechanisms that lead to a higher prevalence of mental comorbidity and health burdens in patients with low SES include behavioral and lifestyle factors such as tobacco abuse, unhealthy diet and insufficient physical activity, lower health literacy, and delayed or non-use of health services,^27^ but also limited access to the health care system. In addition, patients with low SES are more likely to be exposed to negative psychosocial factors.^28^

The paucity of studies addressing the association between SES and psychological comorbidity in cancer means that there is insufficient evidence to support the tailored implementation of patient-centered psycho-oncology support services for patients with low SES. Although numerous problems are cumulative in patients with low SES, such as lower compliance, poorer health literacy and a higher risk of comorbidity and mortality, this patient group is often underrepresented in research. Summarizing the current state of research, there is an urgent need to consider SES alongside other biopsychosocial determinants of psychological comorbidities in cancer patients.

We therefore aimed to provide the 4-week prevalence estimates of mental disorders according to DSM-5 in newly diagnosed cancer patients in relation to their SES, taking into account further demographic and cancer-related factors including level of impairment.

## Patients and methods

### Study design and participants

We conducted a prospective multicenter observational study. Here, we report patients’ data from the first measurement point within 2 months after the first cancer diagnosis. Patients were eligible to participate if they were aged ≥18 years, had a confirmed diagnosis of a malignant solid tumor according to their medical records, were scheduled for cancer treatment at one of the participating cancer centers, had sufficient understanding of German and were physically, mentally and cognitively able to participate. Individuals were not eligible if they had a diagnosis of a second tumor or tumor recurrence.

### Procedures

Between April 2020 and July 2022, potential participants were recruited through the Comprehensive Cancer Centers at University Medical Centers and collaborating hospitals in Germany (Leipzig, Berlin, Hannover, Dresden, Göttingen and Braunschweig). Potential participants were screened for eligibility by study staff using medical records, and received comprehensive verbal and written information about the study. As not all patients could be contacted at the cancer center due to the legal restrictions during the corona pandemic, some received a letter at home with all study information. After obtaining written informed consent from the patients, the study staff made an appointment with the patients to conduct the Structured Clinical Interview for DSM-5 Disorders—Clinical Version (SCID-5-CV)^29^ by telephone.

All participants received a set of questionnaires and were asked to complete them within the next 10 days, either in paper and pencil form or via a personalized link using the LimeSurvey software.^30^ Study staff reminded the participants by telephone after 14 and 21 days. Approval was obtained from the ethics committees of all centers (ethics registration number of the coordinating center Leipzig: 207/19-ek). The study was registered in the International Clinical Trial Registry (No. NCT04620564). The full study protocol has been previously published.^31^

### Measures

Sociodemographic information was gathered using standardized questionnaires during the SCID-5 telephone interview, and medical characteristics using medical records.^31^ We calculated patients’ SES on the basis of three indicators: school education and vocational qualification, income and occupational status.^32^ These initial variables are converted into scales with seven categories each and assigned point values from 1 to 7. The index can assume corresponding values from 3 to 21. In the event of a missing value for a variable, it can be replaced by the arithmetic mean of the other two values. In the present study, the average income of households in 2022/2023 (∼€4000) was used as the basis for assigning income points.

We used the SCID-5-CV^29^ to assess the 4-week prevalence of mental disorders based on the DSM-5 by asking 7 of the 10 modules: (i) mood episodes and persistent depressive disorders, (ii) differential diagnosis of mood disorders, (iii) substance use disorders, (iv) anxiety disorders, (v) obsessive compulsive and related disorders and posttraumatic stress disorder (PTSD), (vi) screening questions on other disorders and (vii) adjustment disorders. For quality assurance, each interviewer received mandatory standardized training in the use of the SCID-5-CV. Test interviews were conducted, one of which was video-recorded and evaluated by a certified psychotherapist. Each interviewer had to confirm that the SCID-5-CV had been carried out correctly.

We used the comorbidity questionnaire modified according to Bayliss et al.^33^ to assess the level of physical morbidity and impairment. On a scale from 1 (‘not at all’) to 5 (‘very much’), patients indicate whether they currently suffer from one of the following 18 comorbidities and the extent to which this affects their daily activities: hypertension, asthma, lung disease, diabetes, thyroid disease, chronic back pain, rheumatism, osteoarthritis, osteoporosis, colon problems, stomach problems, kidney disease, sensory disturbance, heart disease, stroke, neurological disorders, eye disease and mental disorders were asked about. The sum of the conditions, weighted according to the degree of the respective condition, results in the total score, which can range from 0 to 90.^34^

We used the Karnofsky Performance Status Scale^35^ to rate the ability of a patient to carry out usual activities on a score ranging from 0 to 100, where 0 is ‘dead’ and 100 is ‘normal, no complaints, and no signs of disease’.

### Statistical analysis

The sample size is based on the estimated prevalence of mental disorders of 30%. A reliable prevalence estimate should be possible for each SES subgroup. For this purpose, a 95% confidence interval (CI) of 10 percentage points is defined as a sufficiently precise estimate (*N* = 300 per SES subgroup at the last measurement time). In consideration of the longitudinal design of the overall study, which anticipates a drop-out rate of 25% per measurement time point, a base sample of *N* = 2000 patients was calculated.^36^ However, only the data from the initial measurement time point are presented in this paper.

Non-responder analyses were carried out on relevant variables, i.e. age, gender, SES, medical characteristics and performance status, using the chi-square and *t*-tests for patients who refused to participate in the study, and for patients who had to be excluded due to insufficient information.

Frequencies and 4-week prevalence rates were estimated for mental disorders (number of patients, percentage and 95% CI) as raw values and weighted by SES to compensate for over- and under-sampling of SES groups in our sample. For the stratification of the total sample, a ratio of 20% low SES, 60% middle SES and 20% high SES was selected, which corresponds to the SES distribution in the German adult population.^36^ Each case was assigned a design weight (SES prevalence in the general population/SES prevalence in the sample) to account for unequal distribution of the data. Subsequently, survey-weighted means and CIs for mental disorders were calculated using the R package ‘survey’.^37^

To identify sociodemographic and medical predictors for SES-weighted mental disorder, a multistep approach was used. Relevant factors were identified in separate univariate logistic regression models and were then entered in a multiple logistic regression model with ‘any mental disorder’ as the dependent variable. When comparing different tumor entities, breast cancer was chosen as the reference category due to its prevalence rate for any mental disorder closely approximating the overall prevalence rate. Effect sizes were reported as odds ratios (ORs) and their 95% CIs.^38^ Two-sided *P* values < 0.05 were considered significant.

## Results

### Participants

**Figure 1:**
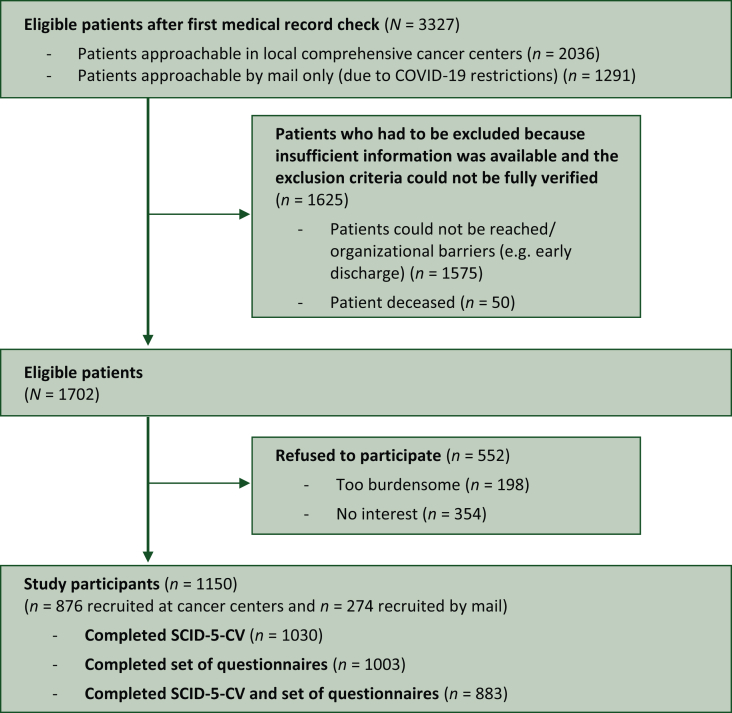
**Flowchart of the sample.** COVID, coronavirus disease; DSM-5, Diagnostic and Statistical Manual of Mental Disorders, Fifth Edition; SCID-5-CV, Structured Clinical Interview for DSM-5 Disorders—Clinical Version.

Out of newly diagnosed patients with malignant solid tumor, we identified a total of 3327 patients to be eligible after medical record check. Due to patient safety restrictions imposed by the hospital authorities during the coronavirus pandemic, 2036 patients could be approached in person by study staff on the ward to provide the study information and verify the other inclusion criteria as required by the study protocol.^31^ There were therefore 1575 patients who we tried to contact in person or by mail, but for whom it was not possible to adequately check the inclusion and exclusion criteria (e.g. language ability). The reasons for this were, for example, early discharge, transfer to another hospital or lack of overall response. Of the remaining eligible 1702 patients, 1150 (67.6%) participated, of whom 1030 had completed SCID-5-CV (Figure 1).

Sociodemographic and medical characteristics are shown in Table 1. We found a higher proportion of men with high SES compared to women (men: high = 44.5%, medium = 40.9 %, low = 14.6%; women: high = 34.4%, medium = 46.5%, low = 19.1%; *P* = 0.003) and a higher proportion of patients with low SES in patients without partnership compared to those with partnership (no partner: high = 22.5%, medium = 43.5 %, low = 34.0%; partner: high = 44.3%, medium = 43.6 %, low = 12.1%; *P* < 0.001).

**Table 1:** Sociodemographic and medical sample characteristics of participants (*n* = 1030)

	%	*n*
**Demographic characteristics**		
Age, years mean (SD, range)	60.2 (13.2, 19-92)	
Sex		
Men	53.2	548
Women	46.8	482
Marital status		
Married	64.8	662
Single	17.1	175
Divorced/separated	11.9	121
Widowed	6.2	63
Partnership	80.2	808
Having children	79.6	813
Educational qualification		
University degree	42.1	432
High school/vocational training	54.5	560
No qualification	3.4	35
Occupation		
Employed	49.9	508
Retired	44.8	457
Unemployed	2.6	27
Other	2.6	27
Socioeconomic status		
Low	16.7	172
Medium	43.5	448
High	39.8	410
Religious affiliation		
Protestant	22.0	226
Catholic	4.9	50
Other	3.9	40
None	69.2	709
Nationality		
German	96.3	989
Other	3.7	38
Residential areas		
Urban (≥20 000 inhabitants)	56.8	583
Rural (<20 000 inhabitants)	43.2	444
**Medical characteristics**		
Tumor site		
Melanoma (C43-C44)	18.8	194
Prostate (C61)	17.2	177
Digestive organs (C15-C26)	15.3	158
Female genital organs (C51-C58)	13.1	135
Breast (C50)	12.5	129
Kidney/urinary tract (C64-C68)	7.3	75
Head and neck (C00-C14)	5.2	54
Lung (C34)	3.0	31
Other	7.5	77
Months since diagnosis^a^, mean, median (range)	1.4, 1.0 (0-6)	
≤2 months	86.8	894
>2 months	13.2	136
UICC		
I	42.8	441
II	21.5	221
III	16.8	173
IV	12.7	131
Not determinable^b^	6.2	64
Cancer treatment received		
No	2.5	26
Yes	97.5	1004
Type of cancer treatment^c^		
Surgery	87.3	876
Radiotherapy	16.0	161
Chemotherapy	20.6	207
Other	12.0	120

Displayed % (*n*), if not otherwise noted; *n* are valid answers only, with deviations from the full sample size being missing values; percentages are based on valid answers.

COVID, coronavirus disease; SD, standard deviation; UICC, Union for International Cancer Control disease stage.

a Months since diagnosis in relation to first questionnaire completion. >2 months: deviation from the study protocol (maximum up to 6 months), since recruitment during COVID-19 was only possible indirectly via mail, which extended the time required for the study inclusion process.

b Not determinable, e.g. in basalioma.

c Multiple response possible; based only on patients who received a cancer treatment (*n* = 1004).

### Non-responder analyses

Patients who were excluded because of insufficient eligibility information (*n* = 1575) were older (mean age 63.4 versus 60.4 years, *P* < 0.001), more likely to be male (58.0% versus 53.9%, *P* = 0.04) and differed in cancer entities (*P* < 0.001) from participants (*n* = 1150). Insufficient data for analyses in SES, Union for International Cancer Control and performance status (Karnofsky scale) were available in this subgroup.

Patients who refused to participate (*n* = 552) were older (mean age 65.9 versus 60.4 years, *P* < 0.001), had a worse performance status (73.6 versus 80.0, *P* < 0.001) and were more likely to have low SES (low SES: 27.4% versus 16.6%, high SES: 26.8% versus 38.9%, *P* < 0.001) compared to study participants (*n* = 1150). No significant differences were observed in gender (*P* = 0.49) and tumor type (*P* = 0.24).

### Four-week overall prevalence estimates

Any mental disorder was diagnosed in 19.9% of patients (Table 2). The most prevalent were depressive disorders (9.2%), trauma and stress-related disorders (6.3%) and anxiety disorders (4.0%).

**Table 2:** Prevalence rates of current mental DSM-5 disorders—raw values and weighted by socioeconomic status

	Raw values *n* = 1030			Weight by SES *n* = 1030		
	%	*n*	95% CI	%	*n*	95% CI
Any mental disorder	19.9	205	(17.6-22.4)	20.9	215	(18.1-23.6)
Depressive disorders	9.2	95	(7.6-11.2)	9.9	102	(7.9-11.9)
Major depression	8.2	84	(6.6-10.0)	8.8	90	(6.9-10.7)
Persistent depressive disorder	1.5	15	(0.9-2.4)	1.6	16	(0.7-2.4)
Bipolar disorders	0.5	5	(0.2-1.2)	0.5	5	(0.0-1.0)
Anxiety disorders	4.0	41	(2.9-5.4)	4.2	44	(2.9-5.6)
Panic disorder	1.3	13	(0.7-2.2)	1.4	15	(0.6-2.2)
Agoraphobia	0.3	3	(0.1-0.9)	0.3	3	(0.0-0.7)
General anxiety disorder	2.3	24	(1.6-3.5)	2.4	24	(1.4-3.4)
Social anxiety disorder	0.9	9	(0.5-1.7)	0.8	9	(0.2-1.4)
Trauma- and stressor-related disorders	6.3	65	(5.0-8.0)	6.3	65	(4.7-7.9)
Posttraumatic stress disorder	1.8	18	(1.1-2.8)	1.7	18	(0.9-2.6)
Adjustment disorder	4.7	48	(3.5-6.1)	4.7	49	(3.3-6.1)
Obsessive compulsive disorder	0.6	6	(0.3-1.3)	0.6	7	(0.0-1.2)
Substance use disorders	3.7	38	(2.7-5.0)	3.6	37	(2.4-4.8)
One mental disorder	16.0	165	(13.9-18.3)	16.7	172	(14.2-19.2)
Two or more mental disorders	3.9	40	(2.9-5.3)	4.2	43	(2.8-5.5)
Screening questions^a^						
Insomnia/hypersomnia	17.6	181	(15.4-20.0)	18.7	193	(16.1-21.3)
Body dysmorphic disorder	4.7	48	(3.5-6.1)	4.8	50	(3.4-6.2)
Somatic symptom disorder	4.1	42	(3.0-5.5)	3.8	39	(2.6-5.1)
Specific phobia	5.9	61	(4.6-7.5)	6.7	69	(5.0-8.4)

Prevalence rates of mental disorders according to DSM-5.

CI, confidence interval; DSM-5, Diagnostic and Statistical Manual of Mental Disorders, Fifth Edition; SES, socioeconomic status.

a Only screening questions for mental disorders and not the full diagnostic criteria applied.

### Four-week overall prevalence estimates weighted by SES

When weighted according to the SES distribution to account for over- and under-sampling of SES groups, 20.9% of patients were diagnosed with a mental disorder (Table 2). At 9.9%, depression is the most common comorbidity, followed by trauma and stress-related disorders (6.3%) and anxiety disorders (4.2%). Insomnia or hypersomnia was found in 18.7% of patients in response to the screening questions on mental disorders.

### Predictors for SES-weighted prevalence of mental disorders

For the following analyses, SES-weighted prevalence rates for any mental disorder were used. Univariate logistic regression analyses revealed significantly higher proportion of patients with any mental disorder in patients younger than 60 years (29.0% versus 14.6%; OR 0.42, 95% CI 0.31-0.57; *P* < 0.001), in women (25.8% versus 16.1%; OR 1.81, 95% CI 1.34-2.47; *P* < 0.001), in patients without a partner (30.8% versus 18.4%; OR 1.97, 95% CI 1.40-2.75; *P* < 0.001) and in those with a higher level of comorbidity and impairment (OR 1.03, 95% CI 1.01-1.05; *P* < 0.001) (Table 3). A significantly higher proportion of women with a tumor in female genital organs had any mental disorder compared to the reference group of breast cancer patients (39.3% versus 21.1%; OR 2.42; *P* < 0.001). A significantly lower proportion of patients with prostate cancer had any mental disorder compared to the reference group (9.5% versus 21.1%; OR 0.39; *P* < 0.008).

**Table 3:** Predictive sociodemographic and medical characteristics on SES-weighted mental disorder

	Proportion of patients with any mental disorder		Univariate regression model		Multivariate regression model^a^	
	%	*n*	OR 95% CI	*P*	OR 95% CI	*P*
Age			0.42 (0.31-0.57)	<0.001	0.42 (0.30-0.59)	<0.001
<60^b^	29.0	130				
≥60	14.6	85				
Sex			1.81 (1.34-2.47)	<0.001	1.22 (0.79-1.87)	0.37
Men^b^	16.1	85				
Women	25.8	130				
SES						
Low^b^	23.8	49				
Medium	21.2	131	0.86 (0.59-1.26)	0.43		
High	16.8	35	0.65 (0.40-1.05)	0.08		
Residential area			1.22 (0.90-1.66)	0.20		
Rural <20 000 inhabitants^b^	19.0	86				
Urban ≥20 000 inhabitants	22.2	127				
Partner			1.97 (1.40-2.75)	<0.001	1.84 (1.28-2.62)	<0.001
Yes^b^	18.4	144				
No	30.8	69				
Tumor entity						
Breast^c^	21.1	27				
Melanoma	14.2	29	0.62 (0.35-1.11)	0.11	0.74 (0.40-1.39)	0.35
Prostate	9.5	14	0.39 (0.19-0.77)	0.008	0.77 (0.32-1.75)	0.53
Digestive organs	23.2	36	1.13 (0.65-2.00)	0.67	1.64 (0.86-3.11)	0.13
Female genital organs	39.3	56	2.42 (1.42-4.19)	0.001	2.45 (1.41-4.34)	0.002
Kidney/urinary tract	20.2	16	0.95 (0.46-1.88)	0.61	1.49 (0.67-3.23)	0.32
Head and neck	18.1	11	0.83 (0.37-1.77)	0.88	1.06 (0.44-2.41)	0.89
Lung	15.0	6	0.66 (0.22-1.67)	0.41	1.05 (0.33-2.85)	0.93
Other	26.7	20	1.36	0.36	1.70	0.17
UICC						
I^b^	21.9	97				
II	20.3	43	0.91 (0.60-1.36)	0.65		
III	20.7	36	0.93 (0.60-1.42)	0.75		
IV	22.1	30	1.01 (0.63-1.59)	0.97		
Level of morbidity or impairment^d^			1.03 (1.01-1.05)	<0.001	1.05 (1.03-1.07)	<0.001

Logistic regression model on any mental disorder, weighted by SES; significant values marked in bold.

CI, confidence interval; OR, odds ratio; SES, socioeconomic status; UICC, Union for International Cancer Control.

a All relevant factors from univariate regression model (*P* < 0.05).

b Reference group.

c Breast cancer was chosen as reference group, since its prevalence rate for any mental disorder was close to the overall prevalence rate.

d Level of morbidity or impairment by chronic physical comorbidities in daily life, adapted version by Bayliss et al. 2005, higher values indicate higher morbidity or impairment.

**Figure 2:**
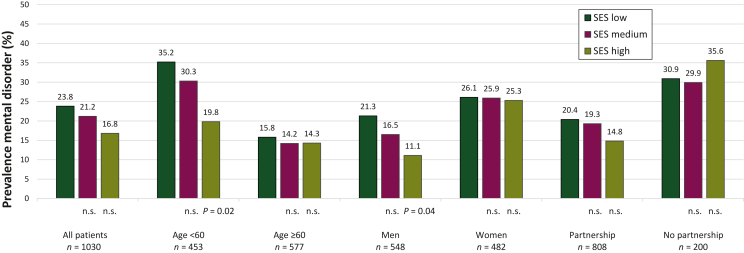
**Prevalenceof any mental disorder stratified by SES in different subgroups.** Any mental disorder assessed via clinical diagnostic interview (SCID-5-CV). Low SES was chosen as the reference group. DSM-5, Diagnostic and Statistical Manual of Mental Disorders, Fifth Edition; n.s., not significant; SCID-5-CV, Structured Clinical Interview for DSM-5 Disorders—Clinical Version; SES, socioeconomic status.

In the total sample, we found no significant difference in any mental disorder between patients with high, medium or low SES. In younger patients (<60 years), the SES was associated with mental comorbidity: patients with low SES had a higher proportion of mental illness than patients with high SES. No such association was found in older patients (>60 years) (Figure 2). Among men, who had a lower overall mental comorbidity than women, patients with low SES had a higher mental comorbidity compared to those with high SES (Figure 2).

Multivariate logistic regression analyses, including all significant predictors of the univariate logistic regression analyses, and weighted on SES, revealed a higher proportion of patients with any mental disorder in patients under 60 years of age (OR 0.42; *P* < 0.001), without a partner (OR 1.84; *P* < 0.001), with a higher level of impairment (OR 1.05, 95% CI 1.03 -1.07; *P* < 0.001) and with a tumor in female genital organs (OR 2.45; *P* < 0.002).

## Discussion

We reported on the 4-week prevalence of mental disorders according to DSM-5 in newly diagnosed patients in the early survivorship phase, stratified by SES for the first time. In summary, we found a total 4-week SES-weighted prevalence of 20.9% for any mental disorder. In the univariate and multivariate analyses, we found no significant differences in the prevalence of mental comorbidity in relation to SES. Multivariate analyses showed that the presence of a tumor in the female genital organs, no partnership, female gender, an age below 60 years and a higher level of impairment best predicted the presence of any mental disorder.

The 4-week total prevalence of 20.9% (95% CI 18.1% to 23.6%) is significantly lower than previous reports, both in comparison to our own study, in which we found 31.8% (95% CI 29.8% to 33.8%),^11^ and in comparison to Singer et al.,^39^ who showed combined prevalence estimates of 32% during acute care (95% CI 27% to 37%). With regard to the most common mental health diagnoses, our prevalence estimates are lower than those reported by Mitchell et al.^10^ for depressive disorders (16.5%; 95% CI 13.1% to 20.3%), for anxiety disorders (9.8%; 95% CI 6.8% to 13.2%) and for adjustment disorders (15.4%; 95% CI 10.1% to 21.6%). However, both of our studies have been remarkable for the precision achieved in estimating prevalences.

There are several explanations for the lower prevalence estimates: Our patients were slightly older on average compared to our previous study,^11^ and the lower psychological distress in older patients is well documented. Our patients were much more homogeneous in terms of initial solid cancer diagnosis and early survival characteristics compared to previous studies. In addition to the exclusion of patients with hematologic cancers, the lower number of patients with advanced disease and higher functional impairment, or a second tumor with a comparatively higher prevalence of mental comorbidity,^10^ may also contribute to a lower prevalence estimate. The relatively high proportion of prostate patients in our sample who have a lower level of psychological distress^11^ could also contribute to the overall lower prevalence. The prevalence of depression and anxiety in our study appears to be generally lower in Germany as a high-income country than in low- and middle-income countries.^40^

Our findings in the early survivorship phase do not replicate the differences in mental comorbidity depending on SES found in other patient groups.^41^, ^42^, ^43^ It is plausible that the psychosocial resources of patients with low SES in this early survivorship phase and in the context of involvement in structured clinical care pathways (where an emphasis on treatment for cure may counter any existential threat) are still sufficient to cope with the stress factors caused by the cancer or cancer treatment. Treatment in comprehensive cancer centers, which offer not only high-quality cancer care but also excellent psycho-oncological care that is free and standard for all patients, could mitigate possible stressors, especially among those with low SES.

Nevertheless, we found two vulnerable groups with regard to higher mental comorbidity, namely younger patients with low SES and men with low SES.^44^ These results confirm previous findings that younger age is a risk factor for mental comorbidity^45^ and that low SES probably has a cumulative effect on mental comorbidity with younger age. In older cancer patients, SES appears to play a subordinate role, which may be explained by successful coping strategies and resilience.^46^

In agreement with previous findings,^44^ our study also shows a gender gap indicating that SES does not seem to play a role in mental comorbidity in women, bearing in mind that in our study women have a lower SES than men, which could have an indirect influence on this result. In contrast, SES in men has a strong influence in that there is a clear difference in mental comorbidity between men with high SES and men with low SES. Higher exposure to negative psychosocial factors, fewer social resources and lower utilization of psychosocial support services are likely responsible for this SES gap in men.

Our multivariate analysis shows that younger patients and those without a partner as well as patients with a tumor in female genital organs and a higher level of impairment have a higher risk of mental disorder. Considering that being single, separated, divorced or widowed significantly increases the risk of an adverse oncological outcome and the likelihood of an earlier cancer death,^47^ our data support the hypothesis that the patients’ single status is also a warning sign for the presence of a psychological comorbidity,^48^ probably triggered mainly by poor social support, but possibly also by premorbid psychological problems that may contribute to being single.

The strengths of our study lie in the multicenter design, the use of SCID-5-CV and the homogeneous sample of first-diagnosed patients at the early survivorship phase. Our study has several limitations. Due to the restrictions imposed by the corona pandemic in all cancer centers, it was not possible to reach all patients who might have been eligible for inclusion in the study. This has the effect of reducing the generalizability of the findings. Furthermore, the original case number plans were not achieved within the study timeframe, resulting in a study that is underpowered. Consequently, it is imperative that we exercise caution in interpreting the results of our study. It is probable that the observed trends in mental comorbidity between patients with low, medium and high SES would have become significant with a larger sample size and correspondingly smaller CIs.

It is also possible that the SES of the sample is slightly overestimated, particularly in the case of multi-person households. The SES calculations are more accurate if income is related to household size, with the weighting being precisely determined by the number of individuals in the household. Nevertheless, it is also necessary to determine the overall financial status, which encompasses the total assets and other sources of income. This is a time-consuming process that has been found to be inaccurate, as a high percentage of individuals do not provide any information on income. The decision to adopt this approach was also influenced by the fact that education is the most significant factor in SES, particularly in relation to health behavior, and is sufficient for the purposes of this research.

With regard to the potential influence of various factors, we lack information on the sexual orientation and migration background of our patients, which could have an impact on the association between SES and mental health. It would be beneficial to investigate these factors in future studies.
